# 94 A Review of the Utility of the Readmission Risk Scale in Hospitalized Burn Injured Patients

**DOI:** 10.1093/jbcr/irad045.067

**Published:** 2023-05-15

**Authors:** Jay Patel, Karen Richey, Claudia Islas, Kevin Foster, Curt Bay, Natalie Kesler

**Affiliations:** AZ Burn Center at Valleywise Health, Phoenix, Arizona; Arizona Burn Center Valleywise Health, Phoenix, Arizona; AZ Burn Center at Valleywise Health, Phoenix, Arizona; AZ Burn Center at Valleywise Health, Cave Creek, Arizona; AT Still Universtity, Phoenix, Arizona; AZ Burn Center at Valleywise Health, Phoenix, Arizona; AZ Burn Center at Valleywise Health, Phoenix, Arizona; Arizona Burn Center Valleywise Health, Phoenix, Arizona; AZ Burn Center at Valleywise Health, Phoenix, Arizona; AZ Burn Center at Valleywise Health, Cave Creek, Arizona; AT Still Universtity, Phoenix, Arizona; AZ Burn Center at Valleywise Health, Phoenix, Arizona; AZ Burn Center at Valleywise Health, Phoenix, Arizona; Arizona Burn Center Valleywise Health, Phoenix, Arizona; AZ Burn Center at Valleywise Health, Phoenix, Arizona; AZ Burn Center at Valleywise Health, Cave Creek, Arizona; AT Still Universtity, Phoenix, Arizona; AZ Burn Center at Valleywise Health, Phoenix, Arizona; AZ Burn Center at Valleywise Health, Phoenix, Arizona; Arizona Burn Center Valleywise Health, Phoenix, Arizona; AZ Burn Center at Valleywise Health, Phoenix, Arizona; AZ Burn Center at Valleywise Health, Cave Creek, Arizona; AT Still Universtity, Phoenix, Arizona; AZ Burn Center at Valleywise Health, Phoenix, Arizona; AZ Burn Center at Valleywise Health, Phoenix, Arizona; Arizona Burn Center Valleywise Health, Phoenix, Arizona; AZ Burn Center at Valleywise Health, Phoenix, Arizona; AZ Burn Center at Valleywise Health, Cave Creek, Arizona; AT Still Universtity, Phoenix, Arizona; AZ Burn Center at Valleywise Health, Phoenix, Arizona

## Abstract

**Introduction:**

The burden of wound care post-hospitalization is often overwhelming, not only for patients and their families but also for home health nurses and intermediate care facilities. Inadequate wound care following hospital discharge can lead to wound deterioration and hospital readmission. These readmissions impact the patient, as well as healthcare institutions, physicians, payor sources, and policy makers. Readmission Risk Scales (RSS) exist but typically focus on those patients with chronic illness. Our hospital’s RRS consists of 16 items: patient without a primary care physician (PCP), active problem list > 2, past medical history > 2, age ≥ 50, lives alone, high fall risk, length of stay > 10 days, mental health disorder, diabetes, heart failure, COPD, alcohol abuse, drug use, uninsured/Medicaid, > 2 ED visits in previous 6 months and prior readmission within 30 days. The purpose of this study was to evaluate the fit of our current RSS for burn patients and identify other variables that may be predictive of readmission in burn patients.

**Methods:**

This was a retrospective chart review of burn patients admitted over a 1-year period. Primary outcome measures were RSS scores, individual RSS variables, social determinants of health and incidence of readmission within 30 days of discharge. Readmitted patients were compared to those who did not require readmission. Descriptive statistics were calculated, and bivariate and multivariable logistic regressions (forward LR) were used to identify variables that contributed uniquely to readmission.

**Results:**

A total of 600 burn patients were admitted over the study period of which 449 met inclusion/exclusion criteria. Thirty-two (7%) of these patients were readmitted within 30 days of their initial hospital discharge. In bivariate analyses, only 4 of the current RRS variables were statistically significant predictors of readmission within 30 days, patient without a PCP (p=.019), active problem list > 2 (p< .001), mental health disorder (p=.004) and drug use (p< .00004). However, when these variables were tested simultaneously in one predictive equation, only drug use remained significantly predictive. The total RSS score accounted for only 1.1% of the variance in readmission within 30 days.

**Conclusions:**

The national readmission rate is 12%, and our overall rate was substantially below this. However, our current Readmission Risk Scale is not predictive in identifying those patients at risk for being readmitted within 30 days of discharge. Further investigation is warranted to identify additional variables to identify at risk patients.

**Applicability of Research to Practice:**

A valid readmission risk assessment tool, would be beneficial to identify patients who are at risk for readmission and enhancing discharge planning.

